# Sclerosing ‘Mucinous’ Blue Nevus: A Clinical Simulator of Dermatofibroma

**DOI:** 10.4137/cpath.s508

**Published:** 2008-03-19

**Authors:** Claudia-S. Vetter-Kauczok, Jürgen-C. Becker, Eva-B. Bröcker

**Affiliations:** Klinik und Poliklinik für Dermatologie, Venerologie und Allergologie, Universitätsklinikum Würzburg, Josef-Schneider-Strasse 2, 97080 Würzburg, Germany

**Keywords:** sclerotic, mucin, nevus

Abundant mucin deposition is an unusual finding for melanocytic lesions. This notion, however, should be toned down since mucin may be present in distinct melanocytic lesions. Indeed, following the first description of mucin depositions in neural differentiated naevi by Maize and Ackerman in 1987 1 this phenomenon was repeatedly observed.^2^,^3^,^4^,^5^ Especially in blue naevi mucin deposition is a peculiar observation. For blue naevi 5 variants are distinguished to avoid the misdiagnosis of melanoma with stromal mucin deposition^6^: (i) neural nevus, (ii) myxoid intradermal nevus, (iii) alveolar cellular blue nevus, (iiii) ancient melanocytic nevus, (v) sclerosing blue nevus.

In 2003, Rongioletti and Innocenzi described the first two examples of sclerosing blue naevi with an abundant mucinous stroma. Herein, we describe the third case of this rare entity. It should be noted, that particularly this uncommon variant of a blue naevus is easily mistaken as desmoplastic-neurotropic melanoma in which the present stromal deposition of mucin is a more typical finding.

A specimen from the right lower leg of a 53-year old man was referred to our dermatopathology department with the clinical diagnosis of a dermatofibroma. Clinically, the tumor appeared as a brown lesion with a regular and sharp border.

The histopathological work up revealed a symmetrical, well circumscribed papule; the fibro-mucinous stroma contained spindle-shaped and dendritic melanocytes, which sometimes were arranged in whorls (Fig. 1A). Orderly arranged melanocytes in the periphery were characterized by pigmentation, as confirmed by melanin-staining. Melanophages were present as clearly seen in the reaction against CD68. Alcian blue staining confirmed the mucin deposition within the tumor and excluded an intradermal component (Fig. 1B). Immunohistochemical staining with Mart-1 (Fig. 1D) and S-100 confirmed both the nature and the distribution of the melanocytes. Notably no reaction was found against MIB-1 excluding a high proliferation rate (Fig. 1C). Thus all criteria for the diagnosis of a sclerosing mucinous blue naevus were fulfilled, and the differential diagnosis of a malignant melanoma was excluded. Moreover, the clinical diagnosis of a dermatofibroma was ruled out by lack of either epidermal changes or typical infiltrating pattern. Hemosiderin deposits which mimic melanin were excluded by special staining.

The cause of mucin deposition in melanocytic lesions remains elusive. It was attributed to a partial involution of the respective lesions. Interestingly, the dermoscopy of the presented sclerotic blue nevus revealed a hypopigmented center; a notion which has been described before.^7^ In line with this hypothesis it is well established that cellularity of naevi decrease which age, whereupon melanocytes are replaced by fibrous matrix, mucin and sometimes fat.^8^

With the present case report we like to raise the awareness for this rare entity because of the momentous differential diagnosis. Further reports of such cases would help to learn more about its clinical, dermoscopic and histological features.

## Figures and Tables

**Figure 1.**
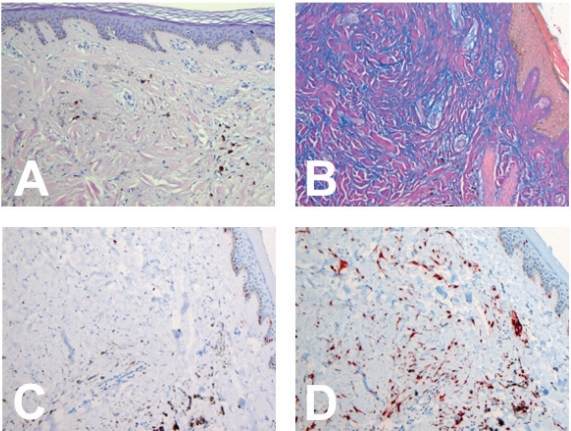
(**A**) Fibro-mucinous stroma with spindle-shaped and dendritic melanocytes, which sometimes were arranged in whorls. Melanophages were present (20x Hematoxylin and Eosin). (**B**) Alcian blue staining confirmed the mucin deposition within the tumor (20x Mucin). (**C**) No reaction was found against MIB excluding a high proliferation rate (20x MIB-1). (**D**) MART-1 staining confirmed both the nature and the distribution of the melanocytes (20x MART-1).
